# Pre-Saturation Technique of the Recycled Aggregates: Solution to the Water Absorption Drawback in the Recycled Concrete Manufacture ^†^

**DOI:** 10.3390/ma7096224

**Published:** 2014-09-01

**Authors:** Julia García-González, Desirée Rodríguez-Robles, Andrés Juan-Valdés, Julia Mª Morán-del Pozo, M. Ignacio Guerra-Romero

**Affiliations:** Superior and Technical School of Agricultural Engineering, University of Leon, Avenida de Portugal 41, 24071 Leon, Spain; E-Mails: desiree.rodriguez@unileon.es (D.R.-R.); andres.juan@unileon.es (A.J.-V.); julia.moran@unileon.es (J.M.M.P.); ignacio.guerra@unileon.es (M.I.G.-R.)

**Keywords:** construction and demolition waste, compressive strength, slump test, moisture state of recycled coarse aggregate

## Abstract

The replacement of natural aggregates by recycled aggregates in the concrete manufacturing has been spreading worldwide as a recycling method to counteract the large amount of construction and demolition waste. Although legislation in this field is still not well developed, many investigations demonstrate the possibilities of success of this trend given that concrete with satisfactory mechanical and durability properties could be achieved. However, recycled aggregates present a low quality compared to natural aggregates, the water absorption being their main drawback. When used untreated in concrete mix, the recycled aggregate absorb part of the water initially calculated for the cement hydration, which will adversely affect some characteristics of the recycled concrete. This article seeks to demonstrate that the technique of pre-saturation is able to solve the aforementioned problem. In order to do so, the water absorption of the aggregates was tested to determine the necessary period of soaking to bring the recycled aggregates into a state of suitable humidity for their incorporation into the mixture. Moreover, several concrete mixes were made with different replacement percentages of natural aggregate and various periods of pre-saturation. The consistency and compressive strength of the concrete mixes were tested to verify the feasibility of the proposed technique.

## 1. Introduction

The great amount of construction and demolition waste generated, and the decline of natural resources for the construction industry, has come to be seen as a worldwide problem and, therefore, many researchers are trying to provide a solution [1,2,3]. Hence, the reuse of construction and demolition wastes (CDW) as a raw material in construction activities, *i.e.*, as a partial replacement of the natural coarse aggregate in the production of recycled concrete is, nowadays, a trend.

Several countries possess legislation governing the use of recycled aggregates in the manufacturing of recycled concrete. Some of them, such as Spain, England, and China [4,5,6], only allow the use of concrete recycled aggregates composed of at least 90% of crushed concrete and natural stone with adhered mortar—albeit this type of waste is not the more commonly produced at work sites. However, other countries, such as Germany and The Netherlands, also include, in their regulations, the use of mixed recycled aggregates, those with varying proportions of crushed concrete, stone with mortar adhered, ceramics and masonry, asphalt, and other materials of stone origin—for concrete production [7,8].

Despite the fact that legislation in this field is still not widespread, scarce standards govern the manufacture of recycled concrete in the majority of countries, there are many scientific investigations [9,10,11,12] studying the characteristics of the recycled concrete obtained with partial to full replacement of the natural aggregate with multiple recycled aggregate options and showing the feasibility of such practices. However, all the studies concluded that the high water absorption of the recycled aggregates is one of the limiting factors in the quality of the recycle concrete obtained, as it has a negative impact on the consistency, the workability, and the mechanical and durability properties of the final product.

In Spain, Annex 15 “Recommendations for using recycled concrete” of the Code on Structural Concrete (EHE-08) [4] regulates the use of recycled aggregates in the manufacturing of recycled concrete. This standard limits the maximum value of water absorption for recycled aggregates up to 7%. However, the majority of concrete and mixed or ceramic recycled aggregates exceed this value. Recycled aggregates from crushed concrete have a higher percentage of water absorption than natural aggregates due to the mortar adhered to the aggregates’ surface, which increases the void ratio of the recycled aggregate [13,14,15,16,17,18]. In the case of recycled aggregates with elevated ceramic content, the water absorption values are even greater due to the nature of the ceramic aggregates with a high clay content and porous crystal structure [19].

The ability of these aggregates to absorb more water directly affects the amount of water available for mixing, which influences the hydration of the cement and the concrete workability in the short-term and the mechanical and durability properties in the long-term. Yang *et al.* showed that slump decreases as the amount of recycled concrete aggregate, used to replace natural aggregate, increases, and that the higher the proportion of ceramic content in the recycled aggregate, the more marked this trend becomes [18,20]. Several studies showed that the porosity of the components used in the manufacturing of concrete results in poorer performance with regards to compressive strength, modulus of elasticity, shrinkage, resistance to freeze-thaw cycles, *etc.* [14,21,22]. Poon *et al.* [23] demonstrated that the initial setting of fresh concrete depends on the content of free water, and also observed changes in the slump measures if some time elapsed after mixing, which corroborate that dry aggregates tend to collect more amounts of free water from the mixture.

Thus, techniques that provide the recycled aggregate with an amount of extra water must be sought in order to solve the porosity problem of the recycled aggregates, and achieve concrete mixes with the *a priori* calculated water/cement ratio. Barbudo * et al.* [24] proposed the use of plasticizers additives as a solution to avoid decreases in the main mechanical properties of the recycled concrete in replacements of 100% of the natural coarse aggregate. Matias *et al.* [25] also support that the use of superplasticizers can compensate for the loss of compressive strength and tensile strength resulting from the introduction of recycled aggregates in concrete. Others authors found solutions without resorting to additives. Rodrigues *et al.* [26] recommended washing the aggregate to remove the fine fraction adhered part of the soluble sulfate content and to decrease the water absorption; Wattanasiriwech *et al.* [27] proposed to adjust the cement content in the concrete mix and the curing conditions. Lastly, some studies [28,29,30] proposed the saturation of the recycled aggregates to prevent the rapid decline in workability shown by recycled concretes. Within this latter group of proposals, the technique proposed in this article seeks to solve the problem of the dry consistency when recycled aggregates are used. The pre-saturation of the recycled aggregate, prior to its addition to the mixture, is one of the fastest and most economical methods.

This paper aims to demonstrate the feasibility of the pre-saturation of recycled aggregates to improve the consistency and workability of the recycled concrete when different percentages of coarse natural aggregate are replaced. In order to do so, first, an optimum time of pre-saturation was assessed to determine the minimum soaking time that enables the recycled aggregate to reach a moisture state that does not compromise the mixing water. In a second stage, several concrete mixes were made with different replacement percentages of the natural aggregate, and various periods of pre-saturation of the recycled aggregates, in order to evaluate the consistency through slump measurements. Finally, and as verification of the adequacy of the applied technique, compressive strength tests were performed to demonstrate that the proposed solution does not decrease the mechanical strength.

## 2. Materials and Methods

### 2.1. Materials

The cement used was Portland CEM III/A 42.5 N/SR (Corporación Masaveu, Oviedo, Spain) and the fine aggregate employed was natural river sand with a granulometric modulus of 3.61. Siliceous gravel with a maximum size of 16 mm and a granulometric modulus of 7.14 was utilized as natural coarse aggregate. The recycled aggregate, which presented a maximum size of 16 mm and a granulometric modulus of 7.67, was supplied by TEC-REC (Tecnología y Reciclado), a construction and demolition waste management plant located in Madrid (Spain).

**Figure 1:**
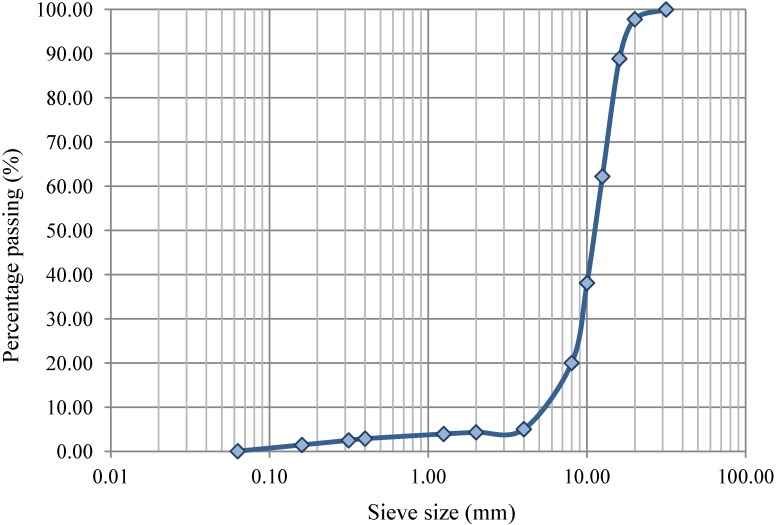
Granulometric curves of the aggregates.

Firstly, the recycled aggregate was tested to verify its compliance with the requirements established in the standard UNE-EN 12620:2003+A1: 2009 [31] and in Chapter VI of the Spanish Code on Structural Concrete (EHE-08) [4], (Table 1, Figure 1).

**Table 1:** Characterization tests of the recycled aggregate.

Standard	Test and result	Limit value [4]
UNE-EN 933-1:2012 [32]	Particle size analysis *D*/*d* ratio: 4	≥1.4
UNE-EN 933-2:1996+1M:1999 [33]	Fines content: 0.04%	≤1.5%
UNE-EN 1097-6:2001+A1:2006 [34]	Apparent density (ρ_a_): 2.53 Mg/m^3^	-
	After oven-drying density (ρ_rd_): 2.08 Mg/m^3^	-
	Saturate surface density (ρ_ssd_): 2.26 Mg/m^3^	-
	Water absorption: 8.53%	≤7%
UNE-EN 1097-2:2010 [35]	Los Angeles coefficient: 40.99%	≤40%
UNE-EN 933-3:2012 [36]	Flakiness index: 14.75%	≤35%

The results showed two non-compliances for the mixed recycled aggregate. Los Angeles value exceeded the 40% limit; however, as it was under the 50% limit, the EHE-08 [4] allows their use in concrete with compressive strength inferior than 30 MPa, such as the concretes tested in the present study. As expected, the water absorption limit was also exceeded.

### 2.2. Concrete Mixtures

Four concrete mixes were prepared using natural aggregate (NA) and recycled aggregate (RA), in the following combinations (Table 2).

**Table 2:** Proportions ofnatural andrecycled aggregateused in the different concrete mixtures.

Mixes	Percentage of NA	Percentage of RA
**Mix 0**	100%	0%
**Mix 1**	80%	20%
**Mix 2**	50%	50%
**Mix 3**	0%	100%

All concretes were calculated to achieve a target mean compressive strength of 25 MPa. The amounts of all the materials incorporated into each mixture are shown in Table 3.

**Table 3:** Components usedin the concrete mixtures.

Components (kg/m^3^)	Percentage of RA			
	0%	20%	50%	100%
**Water**	215	215	215	215
**Water/cement ratio**	0.55	0.55	0.55	0.55
**Cement**	390.9	390.9	390.9	390.9
**Sand**	667.1	669.2	744.1	811.0
**Gravel**	1060.9	790.0	442.6	0.0
**Recycled aggregate**	0.0	197.5	442.6	732.3

**Figure 2:**
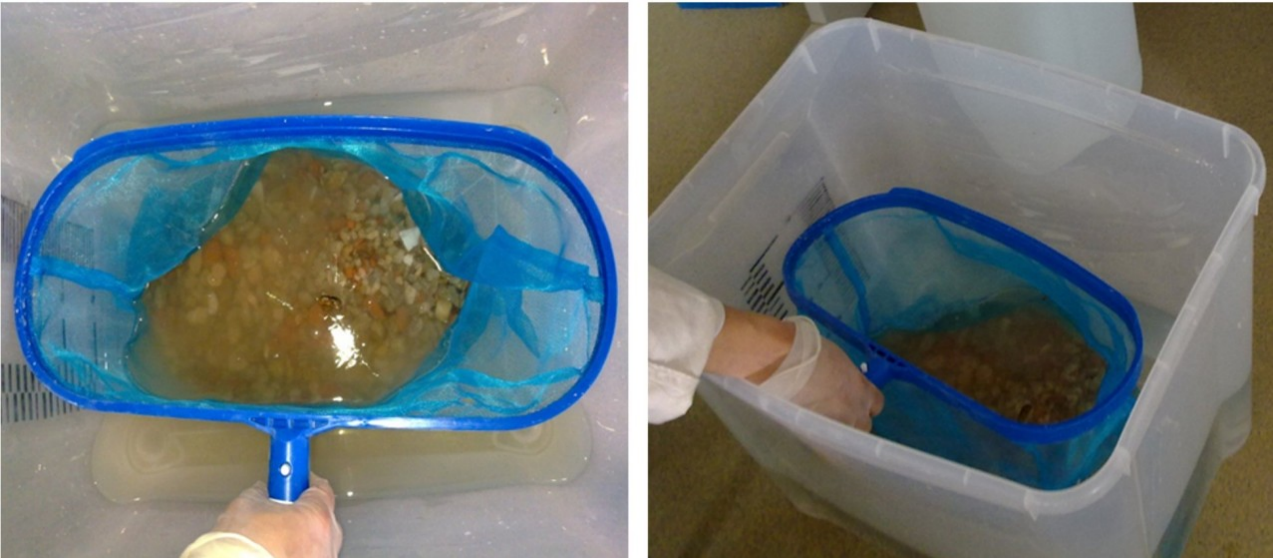
Pre-saturation of recycled aggregate prior to the addition in the concrete batch.

The recycled aggregates used in the different mixtures were previously subjected to the following treatments:

- Air Drying(S).
- Immersedin waterfor a certain “X” period(P-X) (Figure 2).

### 2.3. Pre-Saturation Period

One of the objectives of the pre-saturation is to provide with a technique of rapid implementation, for this reason the soaking period should be as brief as possible, but allow the recycled aggregates to reach an optimum moisture status, thus, when mixed with the rest of components, they do not absorb the mixing water.

To determine the soaking period, a study based on hydrostatic weighing was conducted to assess the time that a sample of recycled aggregate takes to reach a complete state of saturation.

### 2.4. Consistency

**Figure 3:**
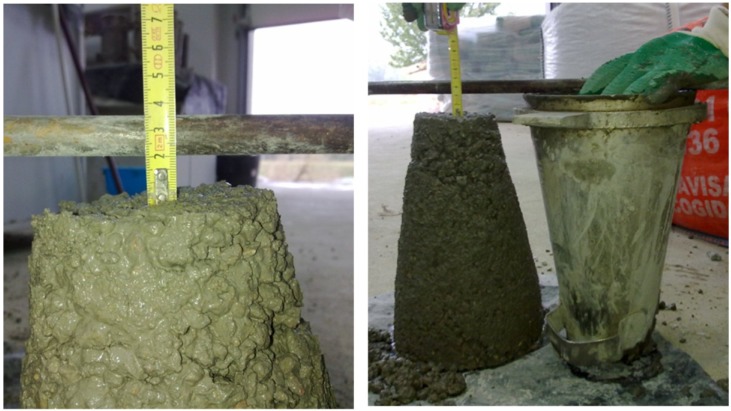
Slump test.

In order to verify the efficiency of the pre-saturation technique a slump test was carried out according to the standard UNE-EN 12350-2 [37], as this is a very simple test to perform on site, requires no expensive equipment or specialized personnel, and provides satisfactory results (Figure 3). Aggregates did not exceed the limit of 40 mm size established by this test, and, thus, it was not necessary to remove any fraction of the coarse aggregate employed.

### 2.5. Compressive Strength

Given that the addition of water to the mixture can result in loss of mechanical strength, a 28-day compressive-strength test was carried out according to the standard UNE EN 12390-3:2009 [38], to ensure that the recycled concrete with pre-saturated recycled aggregates reached the targeted mean compressive strength.

## 3. Results and Discussion

### 3.1. Pre-Saturation Period

The immersion in water, to which the recycled aggregates were subjected to determine the optimum soaking period, showed that mixed recycled aggregates reached the complete saturation after 10 days and that a saturation up to 56% was achieved in the first hour (Table 4).

**Table 4:** Percentage ofwater absorption during the first hour of soaking.

Time (min)	Absorption (%)
0	0.0
3	47.5
5	50.2
60	56.1

**Figure 4:**
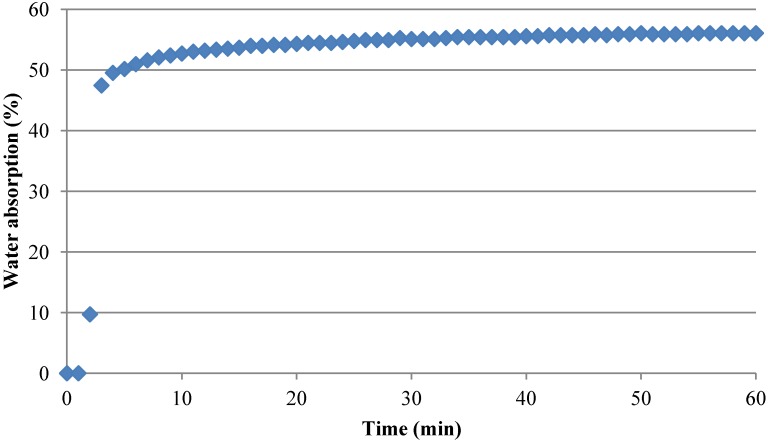
Percentage ofwater absorption during the first hour of soaking.

To determine a brief and effective soaking period, short time intervals with a significant moisture state were sought. Therefore, two times were chosen to be used in the subsequent pre-saturation, a 3 min interval of soaking, in which the recycled aggregate reached up to 47% of complete saturation, and 5 min interval of soaking, with a 50% of complete saturation (Table 4, Figure 4).

Some other studies have considered that, after 10 min of soaking, the recycled aggregates capture up to 70% of the water absorbed in 24 h [16,17]. It should be mentioned that the sample size, the ceramic content and the amount of adhered mortar have a significant influence in the saturation time of the sample [13,19].

### 3.2. Slump Test

**Figure 5:**
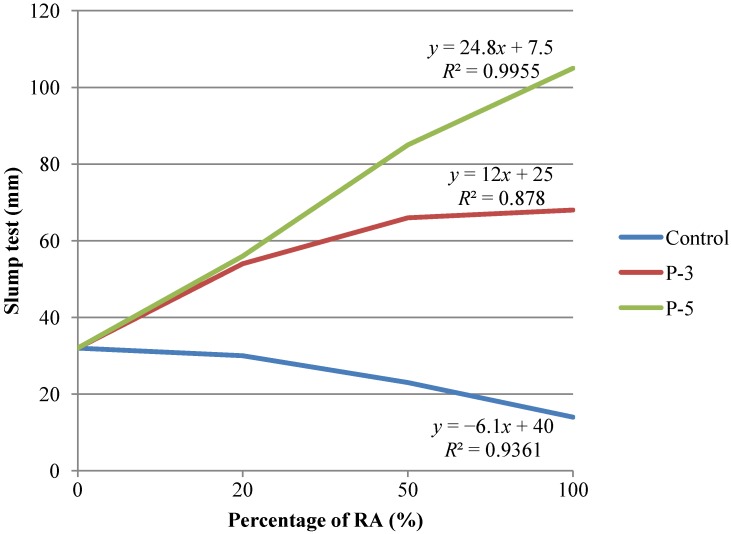
Slump test results for each concrete mix replacement and pre-saturation interval.

The slump test results for each of the concrete mixtures (different percentages of replacement of natural aggregates with recycled aggregates) and the pre-saturation intervals, P-3 (3 min of water soaking) and P-5 (5 min of water soaking) or the control state (air drying aggregates without pretreatment), are shown in Figure 5.

Figure 5 exposes that the pre-saturation of the recycled aggregate caused an enhancement in the consistency of recycled concrete, with increasing slump values as the soaking interval grew. These findings are in consonance with the study of Poon * et al.* [23], which demonstrated the improvement of consistency due to the increase of free water in the concrete mixture when saturated recycled aggregates with dry surface were used, up to 100%-substitution of natural aggregates.

Concretes mixes from the P-3 series achieve plastic and soft slump consistencies for either of the replacement ratios studied, which are the most commonly desired consistencies in the manufacture of concrete. On the other hand, concretes mixes from the P-5 series incorporated a greater amount of water, which resulted in reaching soft or fluid slump consistencies.

### 3.3. Compressive Strength Test

**Figure 6:**
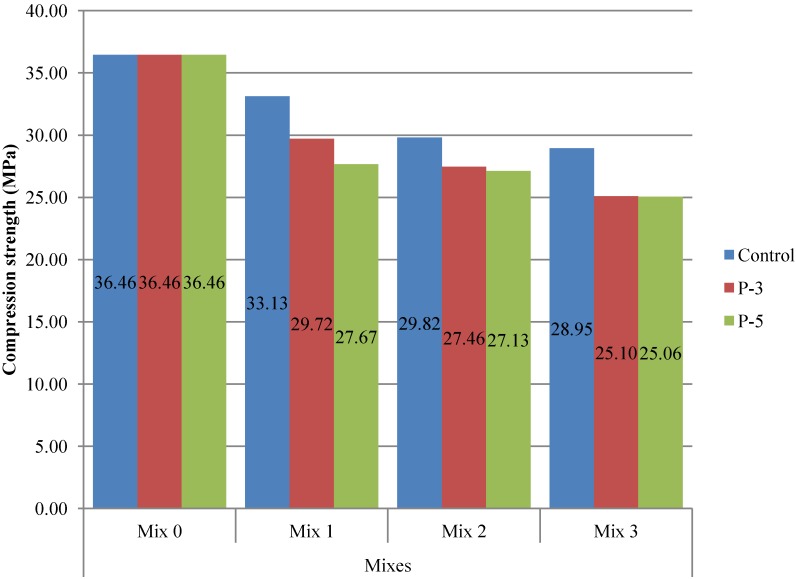
Compressive strength test results.

The compressive strength test results are shown in Figure 6. On one hand, the compressive strength decreased with the pre-saturation interval underwent by the recycled aggregate. Regarding to the foreseeable reduction of mechanical strength caused by the soaking period, concrete mixes from the P-3 series presented a loss, up to 11%, and concrete mixes from the P-5 series presented a loss up to 13% if compared with the control concrete mixes (recycled aggregates without any type of pretreatment).

Similar results were obtained in the investigation of Mefteh * et al.* [39] where recycled aggregates in three different moisture states (dry, saturated, and saturated with a dry surface) were tested. Saturated aggregates and saturated aggregates with a dry surface showed a worse compressive resistance due to the higher water content in the mixtures. When the aggregate is pre-saturated, its ability to absorb water decreases and, therefore, the recycled aggregates absorb less of the initially calculated water for cement hydration. In view of the above, it is considered that the entire amount of water in the mixtures with pretreated aggregates is higher.

On the other hand, compressive strength diminished as the percentage of recycled concrete increased. Two factors are involved in this phenomenon. Firstly, the increase in the replacement ratio of natural aggregates indirectly implied a greater addition of water in the concrete mixture. Secondly, the increase in the replacement ratio of natural aggregates implied a greater addition of recycled aggregates, which possess lower quality (lower bulk density, large amount of adhered mortar content, and weak interfacial transition zone between the aggregate and the cement paste) [12,40] than the conventional gravel. Several authors [41,42] attributed the decrease of compressive strength solely to the latter factor, while other authors [43,44,45] concluded that the increase in the replacement ratio does not negatively affect the compressive strength.

Despite the findings, it is worth noting that the target mean compressive strength of 25 MPa was achieved for all tested batches. Thus, the feasibility of the technique for solving the water absorption problem of recycled aggregates is demonstrated.

## 4. Conclusions

The present study demonstrates the feasibility of the pre-saturation technique in order to solve the water absorption problems of recycled aggregates in concrete manufacture. The results obtained verified that if recycled aggregates are immersed in water for short intervals the consistency of the fresh recycled concrete improved at the expense of an insignificant decrease in the compressive strength.

In view of the results shown in this paper, it was found that the soaking of the recycled aggregate in water for about 3 min prior to the incorporation into the mixture allowed plastic or soft slump consistencies, and that if the pre-saturation period was increased up to 5 min, the slump consistencies reached fluid status. Thus, a more brief pre-saturation technique (P-3) would be sufficient and effective.

The technique mentioned in this article is a quick and easy method to address the problem of water absorption presented by eco-efficient concrete with recycled aggregates. Consequently, the pre-saturation of the aggregates could be adopted in ready mix concrete at the expense of some minor changes in the industrial manufacture process, such as the use of a device to hold the aggregates together while soaking, and that facilitate the draining procedure.

The use of pre-saturated recycled aggregates implied an increase of water in the concrete mixture that leads to a loss in the compressive strength of the recycled concrete. This loss ranged from 11%, for the 3 min soaking period, to 13%, for 5 min pre-saturation interval. However, and despite the strength loss, the target mean compressive resistance was reached in all the samples tested.

Provided that the recycled aggregates meet the requirements of the Code on Structural Concrete EHE-08 [4], and given the similarities between mixed recycled aggregates from different Spanish CDW treatments plants, the soaking intervals presented in this article may be generally accepted.
